# Electrical Resistance Tomography for Visualization of Moving Objects Using a Spatiotemporal Total Variation Regularization Algorithm

**DOI:** 10.3390/s18061704

**Published:** 2018-05-24

**Authors:** Bo Chen, Juan F. P. J. Abascal, Manuchehr Soleimani

**Affiliations:** 1 Engineering Tomography Lab (ETL), Department of Electronic and Electrical Engineering, University of Bath, Bath BA2 7AY, UK; B.Chen@bath.ac.uk; 2 Univ Lyon, INSA-Lyon, Université Claude Bernard Lyon 1, UJM-Saint Etienne, CNRS, Inserm, CREATIS UMR 5220, U1206 Lyon, France; juanabascal78@googlemail.com

**Keywords:** electrical resistance tomography, flow measurements, 4D image reconstruction, total variation (TV) algorithm

## Abstract

Electrical resistance tomography (ERT) has been considered as a data collection and image reconstruction method in many multi-phase flow application areas due to its advantages of high speed, low cost and being non-invasive. In order to improve the quality of the reconstructed images, the Total Variation algorithm attracts abundant attention due to its ability to solve large piecewise and discontinuous conductivity distributions. In industrial processing tomography (IPT), techniques such as ERT have been used to extract important flow measurement information. For a moving object inside a pipe, a velocity profile can be calculated from the cross correlation between signals generated from ERT sensors. Many previous studies have used two sets of 2D ERT measurements based on pixel-pixel cross correlation, which requires two ERT systems. In this paper, a method for carrying out flow velocity measurement using a single ERT system is proposed. A novel spatiotemporal total variation regularization approach is utilised to exploit sparsity both in space and time in 4D, and a voxel-voxel cross correlation method is adopted for measurement of flow profile. Result shows that the velocity profile can be calculated with a single ERT system and that the volume fraction and movement can be monitored using the proposed method. Both semi-dynamic experimental and static simulation studies verify the suitability of the proposed method. For in plane velocity profile, a 3D image based on temporal 2D images produces velocity profile with accuracy of less than 1% error and a 4D image for 3D velocity profiling shows an error of 4%.

## 1. Introduction

In the process of production, transportation and distribution of fluids, in particular in the petroleum industry, multiphase flow is commonly used. The measurement of velocities in different phases is an important factor for safety and for controlling the flow, as is the volume fraction. The flow measurement technique using electrical resistance tomography (ERT) and based on cross correlation has been established for many years. With the fast development of computing power, ERT techniques are growing rapidly. In particular 3D ERT, which is now feasible as a non-invasive detection method, where the interior distribution can be displayed with high speed data collection [1,2]. ERT can be applied to several fields that could benefit from its property of being non-invasive, low cost of the required hardware system and good mobility. In this case, EIT has been applied to many aspects of the medical field, such as, lung [3], brain [4], and breast [5] imaging, and also in the geophysical research applications, for instance in works about the study of infiltration and evaporation processes of porous media using EIT and the magneto-electrical resistivity imaging technique (MERIT) described by Haegel and Zimmermann [6], and the study of moisture flow in cement-based materials [7,8], where in [7], a mortar sample has been used to perform an experiment conducted in order to find out the feasibility of quantitatively monitoring this aspect the using 3D EIT, and authors of [8] investigated the capability of EIT of testing fluids of different flow rates and viscosities. 

Electrical tomography and cross-correlation-based flowmeters are ideally suited for measuring multiphase flows and have been adopted in the past few years [9]. Many applications have been suggested, for example, Gurau and Vassallo [10] proposed the use of cross-correlation to calculate the phase velocity of a two phase air-water flow, from the output signals of hot-film probes, and Saoud and Mosorov [11] utilised cross-correlation with ECT in velocity measurements of gas/solid swirl flows.

We are particularly interested in a two-phase flowing system of liquid and heavy oil. In this case, an oil drop breaks away from the core and sinks to the inner wall of the pipe. With the accumulation of oil dropping down, the pipeline can potentially become blocked. It is expected that the location of such an occurrence inside the pipe can be found using the ability of ERT ‘to see inside’. When the fluid is transported, an average flow speed can be reconstructed with the position of the inclusion. If the sticky oil is attached to the pipe, the flow speed would be gradually slowed down. With the measurement of the velocity profile, this situation could be recognized and amended promptly. The ERT image can also provide information about the location of the oil-phase in the pipeline.

Some of the previous studies about velocity profiles were based on 2D ERT with a dual-plane model, where a double-channel ERT system was applied and cross-correlation between output signals obtained from two electrode channels was used. Deng and Dong [12] designed a dual-plane system for the measurement of gas and liquid bubbly flow. With the extracted eigenvalue of the data from the gas/liquid flow in the vertical pipe, the dispersed phase velocity was measured accurately by Dong and Xu [13]. Wu and Li [14] compared the dynamic distribution and velocity among different flow regimes. However, the flow being measured is a 3D structured object, in other word, 2D modelling and measurement methods are a simplification. Many efforts have also been made for 3D imaging with electrical tomography methods such as ECT [15,16] and ERT [17], however, for many cases, especially ERT, 2D imaging techniques are always considered for practical use due to the features of long operation time, more sensitivity to error and the high complexity of 3D ERT imaging [18]. However, in some special cases, the information in the z-direction is quite important and the simplifications of the 2D method may not be enough to describe what we are expecting, for example, when a 3D visualization of the object is expected, or we want to track the movement of an irregular inclusion inside a flow. In this case, 3D measurement methods are necessary [19].

In this paper we present a new method using a single-channel ERT system in a temporal 2D imaging mode for 2D flow visualization and angular speed, and in temporal 3D ERT (4D) mode for axial velocity measurement. A novel spatiotemporal TV algorithm is adapted for dynamic flow visualization for ERT reconstruction together with a cross-correlation for velocity profile calculation. To assess the proposed method, a series of images were reconstructed with the temporal TV algorithm. Cross-correlation was used afterwards for calculating the flow parameters. In order to analyze the cross-correlated result, the actual speed was measured experimentally and the relative error was calculated and further discussed.

## 2. Method

For a general ERT model, the sensing field is a bounded domain, where electrodes are normally attached to its boundary. The electric field and current flux are generated via electrodes with excitation current applied successively, and voltage data are measured from the boundary of domain from the remaining pairs of electrodes. The conductivity distribution will then be reconstructed via boundary data using a mathematical algorithm, where a forward problem and inverse problem are developed.

### 2.1. Forward Problem

The forward problem of ERT is the process of calculating the voltages via a known conductivity distribution, where a numerical technique, finite element meshing [20], is utilised to discrete the domain of interest and to calculate the potential as a forward solver. In an ERT system, an electrical field is generated via electrodes by injecting an electrical current into the boundary and measuring boundary voltages. Given the electrical conductivity σ and electrical potential *u*, the forward model for ERT is given by Laplace’s equation:

(1) ∇·σ∇u=0

Boundary conditions and electrode model must be considered in a forward model. The boundary condition was introduced due to that the electrodes are inevitably attached to the boundary of the sensing field for a conventional ERT model [21]. The current density is given by (2) with Neumann boundary conditions:

(2) $$\sigma \frac{\partial u}{\partial \hat{n}}=J$$

where $\hat{n}$ is outward unit normal.

To choose an appropriate current *J*, a choice of electrode model must be made. Four different models, the ‘continuum model’, ‘gap model’, ‘shunt model’ and ‘complete model’, have been proposed and investigated previously. The complete electrode model (CEM) combined the first three electrode models, and considering constant potential, contact impedance, and the voltage drop on electrodes, and has been used as a constraint of the forward model equation [22]. Given the CEM equation:

(3) $$u+z_{l}\cdot \sigma \frac{\partial u}{\partial \hat{n}}=\;V_{l}\;$$

In Equation (3), $z_{l}$ is contact impedance, u is the constant potential on electrodes and $z_{l}\cdot \sigma \frac{\partial u}{\partial \vec{n}}$ donates the voltage drop on electrodes.

The expression of driving current is the integral of the current density on the electrode surface. From (2):

(4) $$I_{n}=\int^{\text{​}}\sigma \frac{\partial u}{\partial \hat{n}}ds\;n=1,2,\ldots ,l\;$$

(5) $$I_{gap}\;=\int^{\text{​}}\sigma \frac{\partial u}{\partial \hat{n}}ds=0\;off\;the\;electrodes$$

In Equations (4) and (5), *N* is the number of electrodes and *S* is the electrode surface.

The charge conservation law is applied to ensure the uniqueness of the solution of the EIT forward model [9]:

(6) $$\sum_{n=1}^{N}I_{n}=0\;$$

The forward operator *F* is then defined as a map from the conductivity distribution σ to the measured boundary voltage *u*, that is, F(σ)=u.

### 2.2. Inverse Problem for Flow ERT

The linear inverse problem in ERT can be defined as the recovery of a change in conductivity Δσ from a change in measured boundary voltage  Δu, where  Δu=JΔσ. The Jacobian *J* is computed by the Fréchet derivative of *u* with respect to σ [23]. In this case, a reference boundary voltage $u_{0}$ is available, where $\;u_{0}=F\left(\sigma_{0}\right)$,$\;\Delta u=u-u_{0}$, and $\Delta \sigma =\sigma -\sigma_{0}.$

In flow imaging, the unknown conductivity changes and data are 4D objects. Let’s redefine $\Delta \sigma =\left[{\Delta \sigma }_{1},\ldots ,{\Delta \sigma }_{I}\right]$ and data $\;\Delta u=\left[{\Delta u}_{1},\ldots ,{\Delta u}_{I}\right]$, where $\Delta u=\;\tilde{J}\;\Delta \sigma$, for i=1,…,I, and *I* is the number of temporal frames. It is common to recover each frame independently but this is not optimal, as it does not exploit redundant information across frames. In this case, previous works have defined the inverse problem as follows [24]:

(7) $$arg\underset{{\Delta \sigma }_{i}}{\min}\phi \left({\Delta \sigma }_{i}\right)\;s.t.\;∥{J\Delta \sigma }_{i}-\Delta u_{i}{∥}_{2}^{2}\;\leq \;\delta ,\;\forall \;i=1,\ldots ,I$$

where $\phi \left({\Delta \sigma }_{i}\right)$ is a convex regularization functional that carries a priori information of the unknown conductivity distribution for a single frame.

### 2.3. Spatiotemporal Total Variation Algorithm

In this paper, we propose a spatiotemporal reconstruction framework that exploits regularization. We chose spatiotemporal total variation, as ERT images can be well approximated by a piecewise constant function and consecutives frames are expected to be similar. This allows to exploiting redundant information across consecutive frames. The spatiotemporal total variation problem can be written as follows [25,26]:

(8) $$arg\underset{\Delta \sigma }{\min}∥\nabla_{x,y,z}{\Delta \sigma ∥}_{1}\;+\;∥\nabla_{t}{\Delta \sigma ∥}_{1}\;s.t.\;∥\tilde{J}\Delta \sigma -{\Delta u∥}_{2}^{2}\;\leq \;\delta$$

where first and second terms correspond to isotropic spatial TV and temporal TV functional, respectively, and where Δσ represents a 4D conductivity distribution and $\tilde{J}$ is an augmented Jacobian operating on a acts in a frame-by-frame basis.

The constrained optimization problem (7) can be solved using the split Bregman formulation, which efficiently handled constrained optimization and L1-regularization [27,28]. Using the Bregman iteration, the constrained problem (7) is converted to an iterative scheme:

(9) $${\Delta \sigma }^{k+1}=arg\underset{\Delta \sigma }{\min}∥\nabla_{x,y,z}{\Delta \sigma ∥}_{1}+∥\nabla_{t}{\Delta \sigma ∥}_{1}+\overset{I}{\underset{i=1}{\sum }}\frac{\mu }{2}∥\tilde{J}\;\Delta \sigma -{\Delta u^{k}∥}_{2}^{2}$$

(10) $$\Delta u^{k+1}=\Delta u^{k}-{\;\tilde{J}\;\Delta \sigma }^{k+1}+\Delta u,$$

where (8) is an unconstrained optimization problem and (9) is a Bregman iteration that imposes the constraint iteratively. The cost function in (8) is still hard to minimize given the non-differentiability of the TV functional, but this can be easily done with a splitting technique. Including auxiliary variables allow splitting L1- and L2-functional in such a way that they can be solved in separate steps in an easy manner. Images Δσ are given analytically by solving a linear systems and L1-functional are solved using shrinkage formulae. To perform the split, we include $d_{x}=\nabla_{x},\;d_{y}=\nabla_{y},\;d_{z}=\nabla_{z},\;d_{t}=\nabla_{t}$, so Equation (8) becomes

(11) $$\left({\Delta \sigma }^{k+1},d_{x},d_{y},d_{z},d_{t}\right)=arg\underset{\Delta \sigma ,d_{x},d_{y,}d_{z},d_{t}}{\min}∥{(d_{x},d_{y},d_{z})∥}_{1}+∥{dt∥}_{1}+\frac{\mu }{2}∥\tilde{J}\;\Delta \sigma -{\Delta u^{k}∥}_{2}^{2}\;\;\mathrm{st}.{\;d}_{i}=\nabla_{i}\Delta \sigma ,$$

Constraints in Equation (10) can be handled using the Bregman iteration as above, which leads to the following iterative scheme:

(12) $$\left(\mu {\tilde{J}}^{T}\tilde{J}\;+\lambda \sum_{i=x,y,z,t}\nabla_{i}^{T}\nabla_{i}\right)\Delta \sigma^{k+1}=\mu {\tilde{J}}^{T}\Delta u^{k}+\lambda \sum_{i=x,y,z,t}\nabla_{i}^{T}\left(b_{i}^{k}-d_{i}^{k}\right)$$

(13) $$d_{i}^{k+1}=\max\left(p^{k}-\frac{1}{\lambda },0\right)\frac{\nabla_{i}\Delta \sigma^{k+1}+b_{i}^{k}}{p^{k}},\;\mathrm{for}\;i=x,\;y,\;z$$

(14) $$p^{k}=\sqrt{\sum_{i=x,y,z}{\left|\nabla_{i}\Delta \sigma^{k+1}+b_{i}^{k}\right|}^{2}}$$

(15) $$d_{t}^{k+1}=\max\left(\left|\nabla_{t}\Delta \sigma^{k+1}+b_{t}^{k}\right|-\frac{1}{\lambda },0\right)\frac{\nabla_{t}\Delta \sigma^{k+1}+b_{t}^{k}}{\left|\nabla_{t}\Delta \sigma^{k+1}+b_{t}^{k}\right|}\;$$

(16) $$b_{i}^{k+1}=b_{i}^{k}+\nabla_{i}\Delta \sigma^{k+1}-d_{i}^{k+1},\;\mathrm{for}\;i=x,\;y,\;z,\;t$$

(17) $$\Delta u^{k+1}=\Delta u^{k}+\Delta u-\tilde{J}\Delta \sigma^{k+1}$$

Equation (11) is a linear system that can be solved efficiently using a Krylov solver [25,26,29], such as the bi-conjugate gradient stabilized method, which involves only matrix-vector multiplications. The number of Bregman iterations and other imaging parameters are selected empirically.

### 2.4. Cross Correlation for Velocity Profile

The technique has been adopted to calculate the velocity is pixel-pixel or voxel-voxel cross-correlation for 2D and 3D, which produces the velocity between pixels of a cross-section or voxels from different ‘layers’. Cross-correlation is actually working out the transit time between two output signal vectors generated from pixels or voxels, and velocity calculation would be available with the known distance. From [2,5], in terms of the problem mentioned in this paper, the discrete cross-correlation function of series x and y is defined as follows:

(18) $$R_{xy}\left(\tau \right)=\frac{1}{N}\sum_{n=1}^{N}x\left(n\Delta t\right)y\left(n\Delta t+\tau \right)\;$$

where signals x  and y are corresponds to the characteristic value vectors from two different image matrixes that the cross-correlation applied to. *N* is the length of vectors, and Δt  gives time-step. 

The cross-correlation procedure that has been used in this paper has the following steps:

1. Definition of each pixel or voxel by domain division.
2. The characteristic value extracting from each pixel or voxel (in this paper, the characteristic value is given by an average value).
3. Characteristic value vectors are composed from pixels or voxels.
4. Pixel-to-pixel cross correlation or voxel-to-voxel cross correlation conducted by function (18).
5. Plot the result of cross correlation, and the peak will correspond to the transit time.

The acquired transit time τ corresponds to the peak of the plotted cross correlation function $\;R_{xy}\left(\tau \right)$. For the case of 16 pixels (2D) and 27 voxels (3D), the velocity profile is given by [5]:

(19) $$v=\left[\frac{L_{1}}{\tau_{1}},\frac{L_{2}}{\tau_{2}},\cdots ,\frac{L_{k}}{\tau_{k}}\right]\;$$

where k=16 (2D) or 27 (3D).

**Figure 1:**
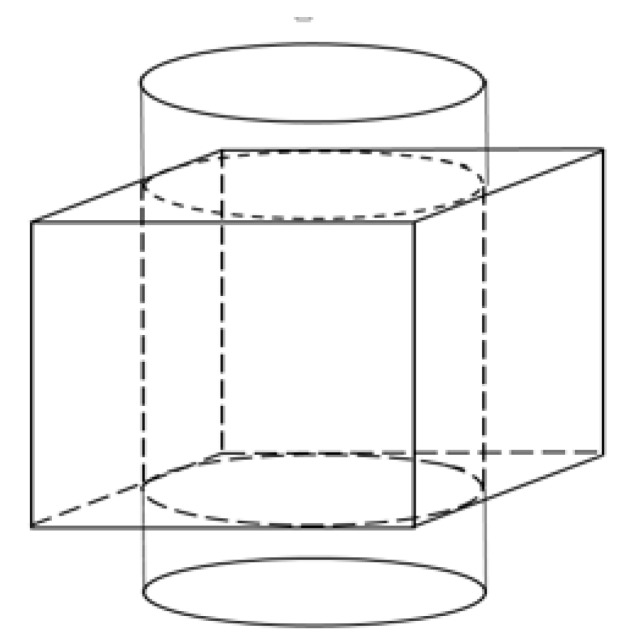
The illustration of the ERT visualization domain within the cylinder tank: the cube indicates the domain has been defined (Jacobian calculation) in this test, where the height of the cube is the distance that will be used for calculating the inclusion moving speed.

In this paper, the average speed of the moving inclusion was calculated and made comparison between different algorithms. In this case, the speed is given by:

(20) $$v=\frac{Height}{m\Delta t}$$

where *Height* is given by the size of *z*-axis of the defined domain, as showing in the Figure 1.

## 3. Results

In this section, the set up and procedure of simulation and experiment in both 2D and 3D situations are demonstrated in details, and the results of each part, including reconstructed images and speed calculation, are displayed and will be further discussed in the following section. The purposes of this section are feasibility analysis on dynamic model monitoring and velocity profile measurement. The proposed temporal TV algorithm will be used for reconstruction with consecutive frames containing time-related information. In addition, pixels and voxels, in both 2D and 3D case, will be defined as a specific area in each frame, where the output signal from each of them is composed with extracted characteristic values from every frames. Moreover, the pixel-pixel and voxel-voxel cross correlation for 2D and 3D cases could be made with these characteristic value vectors in order to work out the speed. Here we describe software flow diagram for simulated and experimental data.

Software process with simulation data:

- Setting up shape and size of the domain, electrode (size, number and locations),
- Selection of excitation and measurement method
- Set up the conductivity value of background ($\sigma_{0}$) and inclusion ($\sigma_{i}$)
- Set up the movement of the inclusion
- Solve boundary data based on FEM
- Jacobian calculation (*J*)
- Image reconstruction based on proposed method
- Using cross correlation to find out the transit time (τ)
- Feasibility analysis of speed calculation (v)

Software process for experimental procedure:

- Phantom design according to the setting up
- Data acquisition ($V_{EX}$),
- Image reconstruction based on proposed method using *J*
- Using cross correlation with thresholding data to find the transit time
- Calculation of the speed of the moving inclusion with the transit time

### 3.1. Simulation in 2D

**Figure 2:**
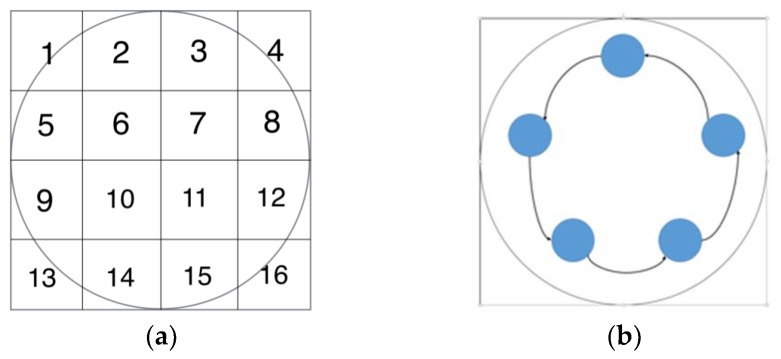
(**a**) The image of pixel unit (**b**) The movement of the object in the simulated domain.

**Figure 3:**
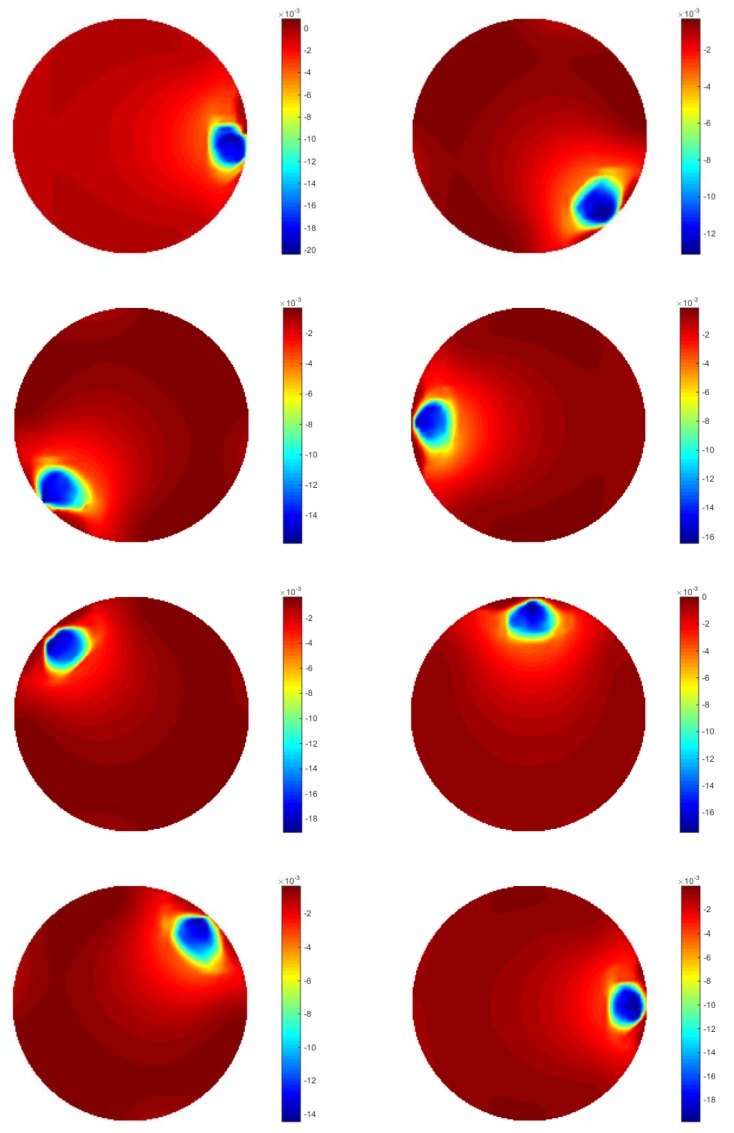
Reconstructed images of inclusion in the simulation test using Temporal TV: The movement path of the inclusion in the simulation test was a circle, where 8 images are selected to show how was the movement of the inclusion within the domain. Each of the sub-graph displaying the location of the inclusion at that time-step.

**Figure 4:**
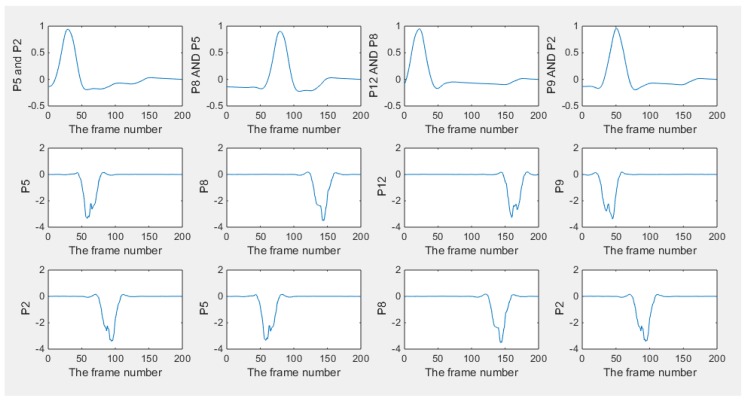
Plots of cross-correlation results and the corresponding pixels in the 2D simulation test: 4 plotted line graphs on the top row represents results of the cross correlation, and the other two plots below each of them shows plots of the characteristic vector that has been utilized for cross correlation. For example, the sub-graph named P5 and P2 means the cross correlation between pixel 2 and 5, and plots of pixel 2 and 5 are displayed below it respectively.

The 2D simulation test was setting up that the inclusion was moving continuously along a circle with a constant angular speed within a 2D circular model, where a 16-electrode ring was applied. The migration path was divided into many steps, where 200 locations were selected homogenously along the path for image reconstruction on each location. The aim of this test is to investigate whether the movement is detectable, and the feasibility of working out the speed. The forward model was constructed using EIDORS, where the injected current was setting at 1 mA, and semi diameter of the sensing field and the movement path are 7 cm and 5 cm with 1.5 cm radius of the circular inclusion. The conductivity of the background and object are 1 and 0, respectively. In order to calculate the velocity distribution through the pixel-pixel cross correlation method, each frame of the image was divided into 16 pixels for characteristic value extracting. The distribution of the pixels is shown in Figure 2a. In Figure 2b, the movement of the inclusion is demonstrated, and some reconstructed images using proposed algorithm are displayed in Figure 3. Some plots of cross correlation result and corresponding pixels are showing in the Figure 4, in which the first four plots on the top represent the cross-correlation result plots, and the ones below show the plots of the corresponding pixels. These plots show the dynamic change of the object movement within each pixel. Satisfactory results are displayed in the top four plotted images and the frame number related with each peak value are consistent with the corresponding two pixel plots below them.

The transit time could be reflected from the frame number of the peak, as shown in Figure 4, where the time-step between adjacent frames is known, and angular speed calculation is attainable. As a result, it is verifying the feasibility of movement monitoring and speed calculating with the proposed algorithm. In the following part, some results from real data will be discussed.

### 3.2. Experiment in 2D

**Figure 5:**
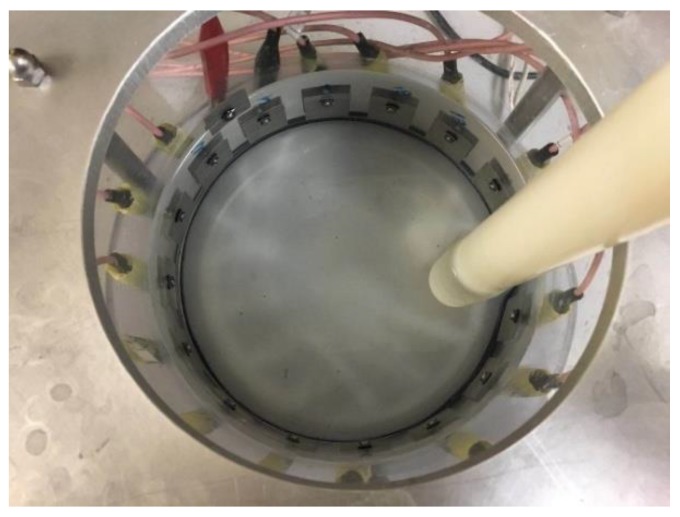
The experimental set up with the 2D ERT tank unit with tap water as a background.

In this part, a series of data were collected from the continuous movement of the inclusion. The phantom adopted (showing in Figure 5), is a cylinder shaped transparent glass container with an inner diameter of 14 cm, and a height of 25 cm. Rectangular shaped stainless-steel sheets (4 cm × 3 cm) were used as electrodes. Screws were utilized for the purpose of connection and fixing on the phantom properly. An extra screw was installed on the wall above the electrode ring to connect with the ground wire with the data collection system. To be consistent with the simulation, the model is kept to be the same size and same way of circular movement. In the 2D experiment, a plastic bar was used as an inclusion and it was moving continuously along the inner wall of the phantom anticlockwise with R = 5 cm until it came back to the origin. There were 70 frames collected from the ERT KHU Mark 2.5 data collection system (Kyung Hee University, South Korea) with an injected current of 100 µA and 11.25 KHz, where neighboring excitation and measurement method were used and 208 individual voltages in total were collected for each frame. The current injection pattern and voltage measurement pattern are summarized in the Table 1. In this case, 70 corresponding images are produced. As mentioned in the 2D simulation, Temporal TV algorithm was used for reconstructing images for proposes of movement monitoring, and angular speed calculation was conducted by cross correlation.

**Figure 6:**
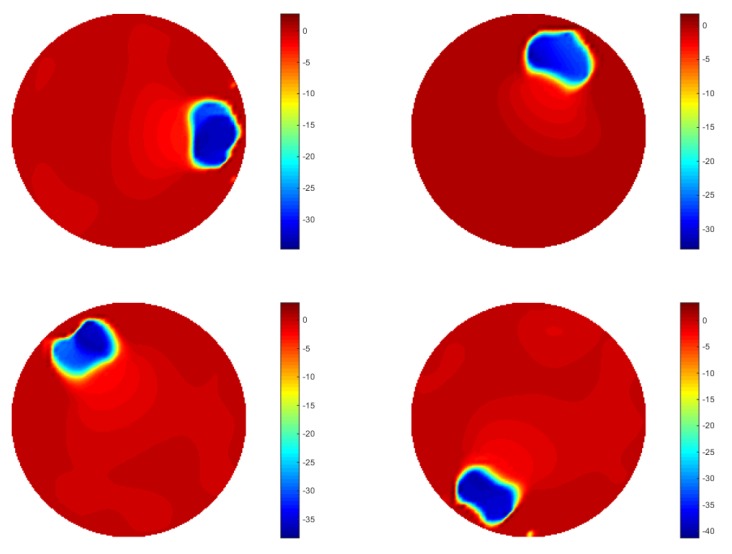

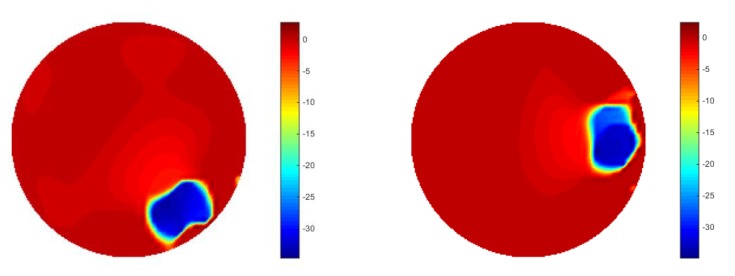
Image reconstruction of 2D experiment using temporal TV: a dynamic image generated in the experiment test, and images displayed here are extracted randomly to show the movement of the sample.

**Figure 7:**
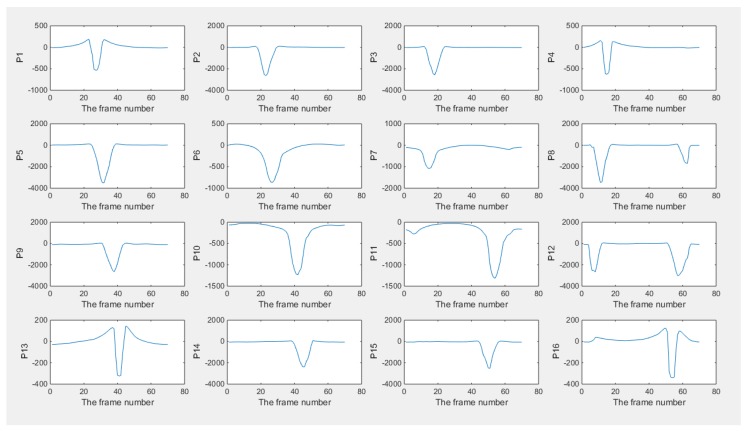
Plots of pixels of the result 2D experimental test with phantom inclusion in the ERT unit: plots of 16 pixels are displayed here, where the peak means that the inclusion entered and left the area of the specific pixel.

**Figure 8:**
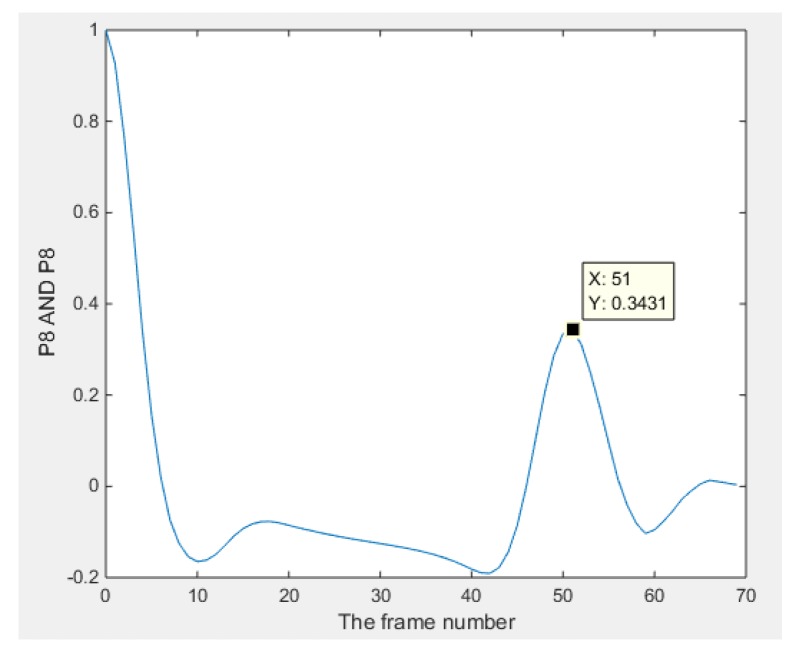
Plots of cross-correlated results using pixel 8.

The pictures in Figure 6 show the result of the reconstruction (only six frames are displayed here for illustrating the monitoring performance). The objects displayed in images got slight bigger and overlapped compared with the case of simulation, which would be caused by the generation of inter-frame data from the dynamically moving inclusion. In terms of the plotted line graphs of 16 pixels (Figure 7), they display that the inclusion is moving from a location within pixel 12, then experienced most of the pixels (can be found from the peak value) along the phantom edge sequentially until it come back again at its origin, which demonstrates the monitoring purpose was achieved. The cross-correlation result is shown in Figure 8, where the peak value indicates that 51 frames were collected while the bar was moving through the whole circle. The transit time is 10.71 s, which could be given by the system data collection speed. Assuming the moving route was a proper circle, the angular speed can be calculated using 360 degrees and the corresponding transit time. The details of the speed calculation and error study are discussed further in Section 4.

### 3.3. Simulation in 3D

In the 3D simulation test, a 16-electrode cylindrical 3D model with two rings of eight electrodes were constructed, where the size of each electrode is 3 cm × 4 cm. Two rings were located at a height of 9 cm and 16 cm, respectively. A neighboring data collecting method was selected for current excitation and voltage measurement. The forward model was built using EIDORS and 3D mesh was generated by NETGEN, where the injected current was setting at 1 mA. The inclusion has been simulated is a sphere with a radius of 1 cm, and the conductivity value was setting at 0 (non-conductive), while we set the conductivity value of the background at 1.

**Figure 9:**
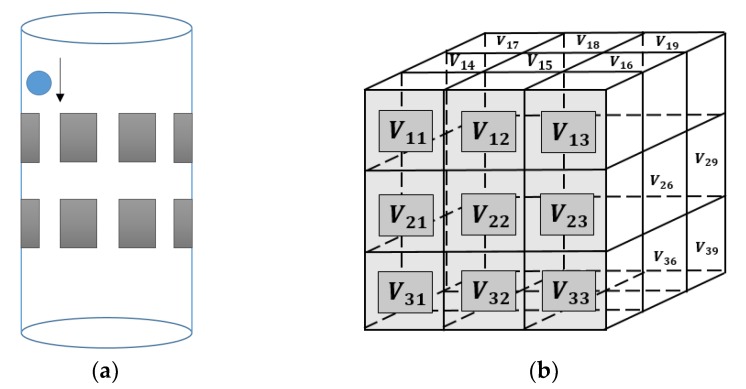
(**a**) The movement of the inclusion in the 3D phantom (**b**) Distribution of 27 voxels within the domain.

In the vertical direction, 71 locations of the inclusion were chosen evenly between (5, 0, 16) and (5, 0, 9), where it was assumed that the sphere was dropping 0.1 cm each step, and a frame (208 independent measurements) of the image is produced split into 27 voxels (3 × 3 × 3). A drawing of the experimental phantom and voxels distribution is shown in Figure 9a,b respectively. The cross-correlation procedure for 3D is similar to the 2D one. For all voxels, a vector of length 71 (number of frames) could be composed by 71 average values from each of the frames, and there were 27 vectors generated in total. Frames are time-related with each other, and the data collection speed of the ERT system and the overall velocity of the movement are both constant, hence, 27 signals are generated. With the cross-correlation method, the transit time between two different voxels comes to be available from signals of different voxels, and the overall flow speed could be calculated with distance.

**Figure 10:**
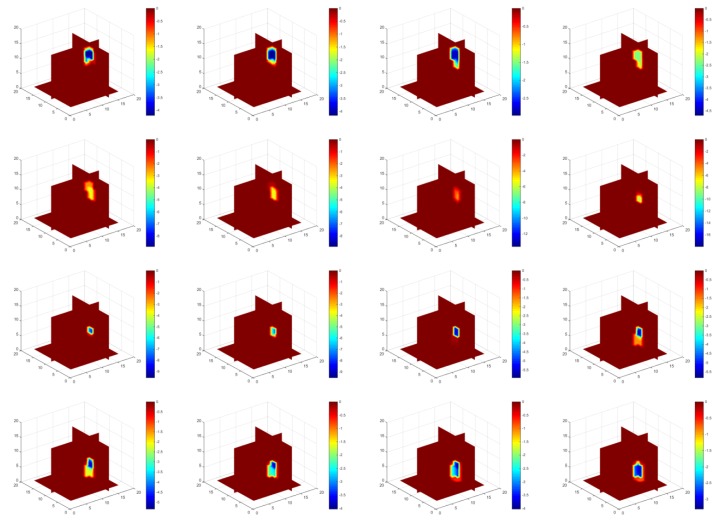
Image reconstruction of 3D simulation using temporal TV: images displayed here are illustrates movement from the top to bottom, which is consistent with the set up of 3D simulation test.

The aim of this test is to find out the feasibility of monitoring and velocity measurement in a 3D model. Based on the simulation set up described above, 3D image reconstruction was conducted by the temporal TV algorithm. The reconstruction results are shown in Figure 10, where the overall situation of the sphere movement was being monitored as expected as the object is moving down. We can see that the object is moving down gradually with the translocation of the inclusion, which demonstrates the good performance of temporal TV, although the objects in the middle seems weaker than the ones located on the top and the bottom.

Compared with 2D ERT reconstruction, it is obvious that the conductivity value of the object (referring to the color bar) was varying with as the location of the inclusion was going downwards, especially those that are located in the middle close to the center in the visualization domain, are suffer especially from low resolution. When the inclusion is on the top and bottom of the domain, the color of the objects from the images indicates a more non-conductive character of the inclusion, and the character weakens when it came to the middle area, although the inclusion was always the same. The reason of this appearance could be that the intensity of the electric field in the center area is relatively weaker than the area closer to the electrodes where the current is injected. The effect of noise (that could potentially be produced from contact impedance and system measurement error, especially in the experimental test), could weaken the character of the movement, which may lead to less accurate result of cross-correlation in 3D. To eliminate noise, some unexpected measurement errors need to be removed, and thresholding is needed before the cross-correlation, where binary image values were used instead of decimal data. In this case, 0 and 1 will be the only two values to appear in the matrix of the reconstructed images.

**Figure 11:**
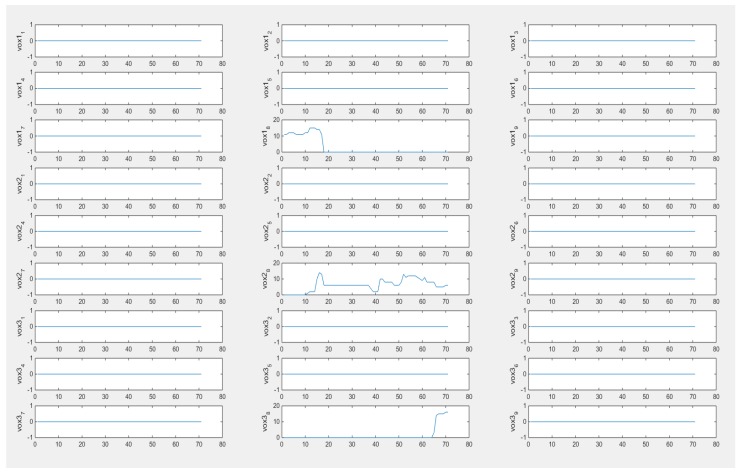
Plots of 27 voxels with thresholding data.

**Figure 12:**
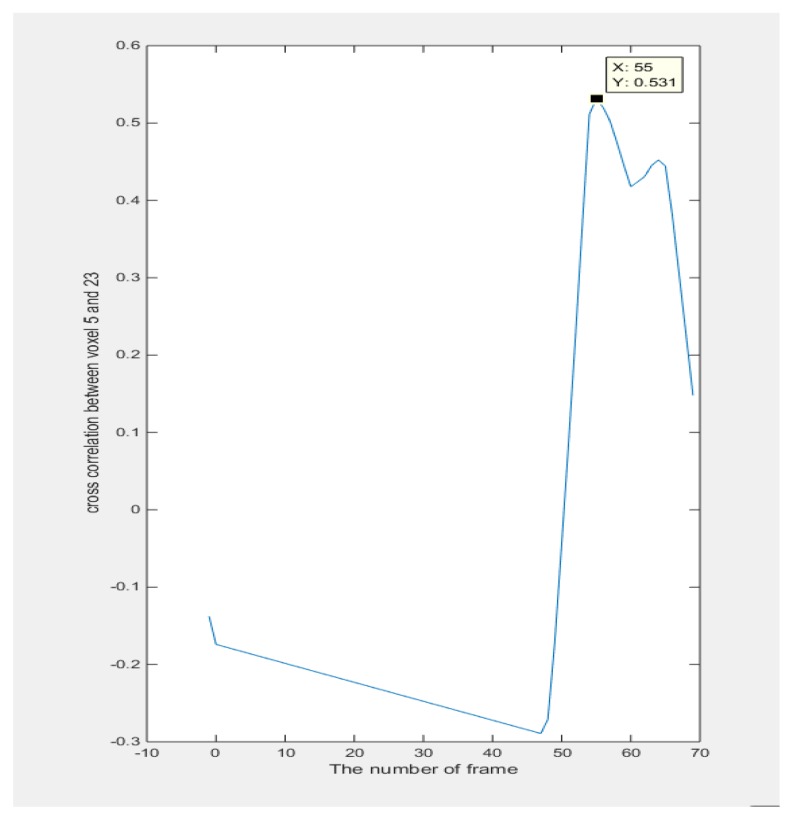
Plots of cross correlation between voxel 8 and 26.

In Figure 11, the output vectors of 27 voxels from 71 frames are plotted, which illustrate the moving process of the object within the simulated phantom. From the plot of vox1_8_, vox2_8_, and vox3_8_, it could be seen that the characteristic value is fluctuating, which could be understood as the process of how the inclusion was entering and leaving the specific voxels. The plots of signals from other voxels are a parallel line and the value remains at 0, which indicates the sample did not go through them and this is consistent with the set up of the simulation test. For cross-correlation of a 3D case, the distance in function (20) is a known constant and determined by the size of height as we defined the sensitivity map (Figure 9b). Considering that the object is moving from vox1_8_ to vox3_8_, the required time could be calculated from the peak value of the cross-correlation plot between these two voxels since the object is moving downwards from vox1_8_ to vox3_8_. Hence, the speed calculation would be achievable. Figure 12 shows the result of the cross-correlation.

This 3D simulation test discussed the performance of sample movement monitoring via 3D image reconstruction of the simulated dynamic movement of the sphere, and speed calculation using the proposed method. It is verified that temporal TV could meet the requirements discussed in this paper. In the next part, some results from real data will be shown, and the calculated speed result will be illustrated and analyzed in Section 4. 

### 3.4. Experiment in 3D

**Figure 13:**
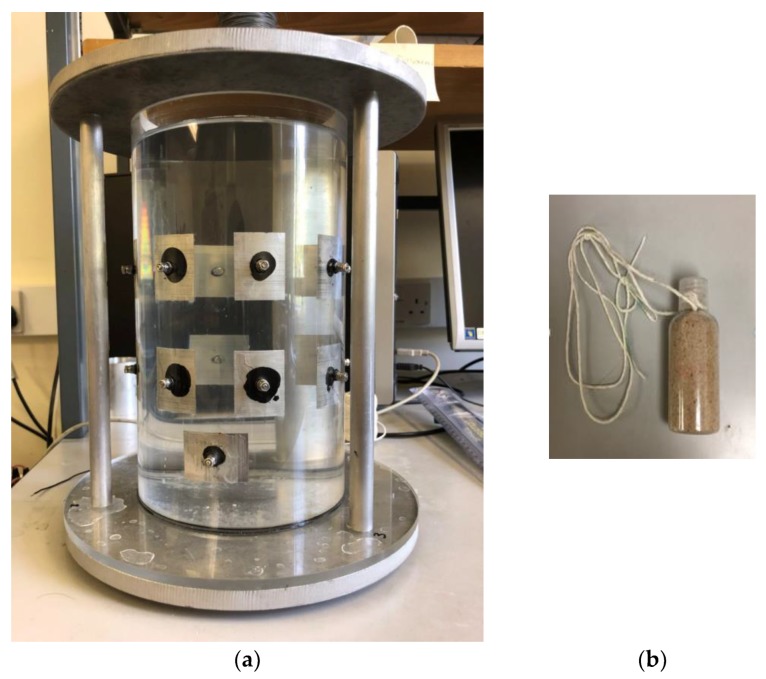
(**a**) Phantom of the 3D experimental test: 16 electrodes composed with 2 rings of 8 are utilized for excitation and measurement. The stainless-steel boards are applied to the tank as the main part for the pipe to sit on, which avoid the problem of leaking. (**b**) Non-conducting inclusion of 3D experiment test: a plastic bottle filled with sands to make sure the inclusion could sink down to the bottle, where tap water was chosen as the background and continuous phase.

The phantom used for the 3D experimental test is designed as a 16-electrode cylinder container with a height of 25 cm and diameter of 14 cm and using the same material with all components as the 2D one. Two rings of eight electrodes (3 cm × 4 cm rectangular-shaped) are fixed on the wall of the sensor at a height of 9 cm and 16 cm, respectively, which is consistent with the situation of the simulation test. A small plastic bottle filled with sand was used to stand in for a dispersed phase and tap water was poured into the phantom as a continuous phase. To create a relative movement, the bottle was controlled to move up and down continuously using a string that connected with the bottle. As shown in the pictures, the bottle was released down and pulled up vertically (starting from the water surface). During the movement, it was on the left-hand side and close to the inner wall of the sensor. The data collection system and method are identical as mentioned before, and the driving frequency was set to be 10 KHz. Figure 13 shows the 3D experimental phantom and the inclusion.

**Figure 14:**
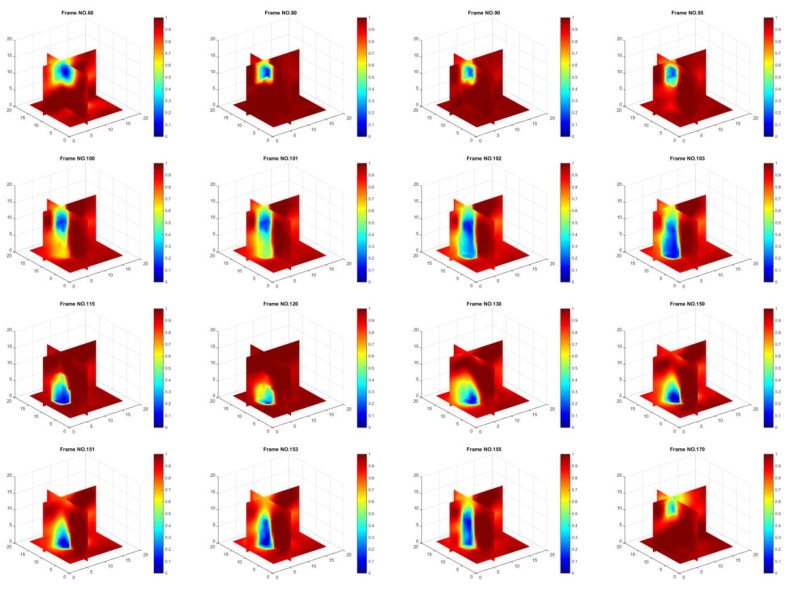
Reconstructions of the movement using temporal TV: a dynamic 3D image was produced, and 16 images selected among 200 frames showing that the movement of inclusion was started from the top move down to the bottom, and come back to top. The Blue object in images (refer to the color bar) corresponds to where the inclusion arrived at that time-step.

During the experiment, there were periods where the bottle movement happened, and 200 frames were collected. The pictures shown in Figure 14 illustrate the whole movement process. Compared with the reconstructed images of the simulation test, it seems that experimental test obtained a slightly degraded result. However, during the simulation, a certain location of the object corresponds to the calculated boundary voltage data, which contributes to a frame of the image. For the 3D reconstruction of the experiment, the real boundary data is measured by a data collection system, and is coming from the dynamic movement of the bottle. It is worth emphasizing that the time required to measure the data of each frame could not be neglected, whereas the simulation did. During this time interval, the bottle was still keep moving, which leads to inter-frame data being produced.

**Figure 15:**
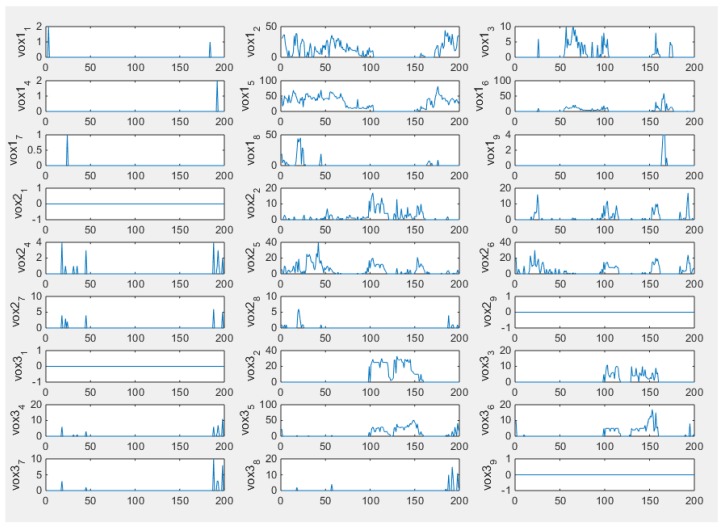
Plots of voxels after thresholding.

The plots of voxels are displayed in the Figure 15, which demonstrate how the movement of the inclusion affects individual voxels. The first few frames could be ignored as the bottle had not started to move, and it is actually the background data which are plotted and would not affect the velocity profile result. Since the real data of the 3D experiment contains more unexpected noise, the reconstructed results shown in Figure 14 are not as good as in the 2D case. To eliminate the unwanted factors, as what was doing in the 3D simulation, thresholding was conducted.

**Figure 16:**
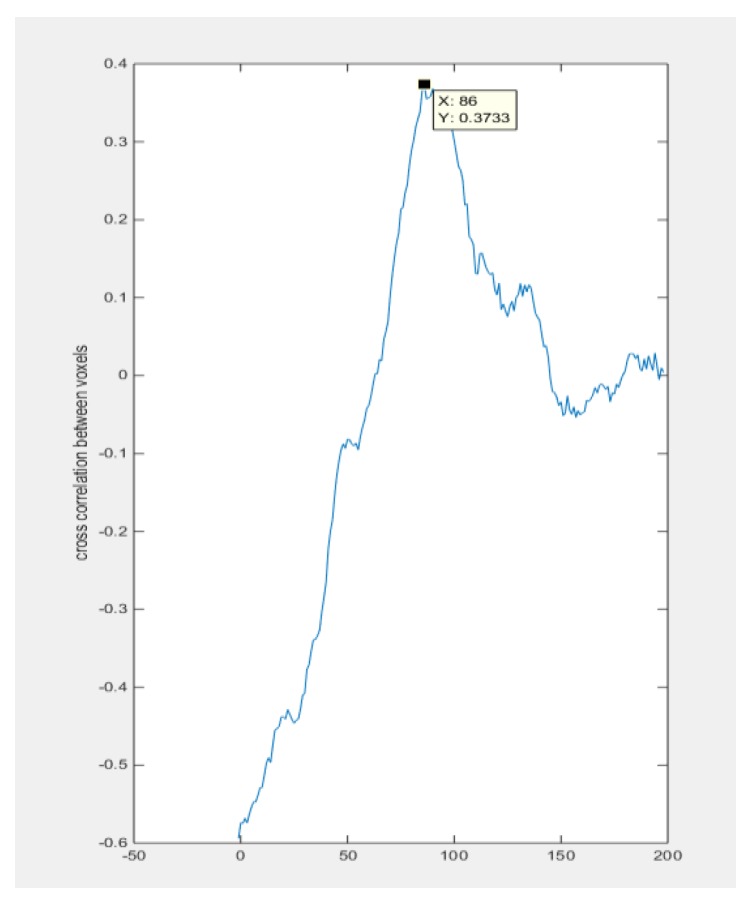
The cross-correlation results: the *x*-axis indicates the frame number, and the *y*-axis gives the value of the cross-correlation result. The peak value on the *x*-axis in this case indicates the transit time of this movement.

The voxels used for the cross-correlation calculation in Figure 15 are from the top voxels layer and bottom voxels layer which correspond to the case that the object was moving through this space. The peak value of the function indicates the moment that two signals have maximum similarity, which gives the transit time of the object moving from the top to the bottom. In the case of Figure 16, the transit time corresponds to 86 frames. The average movement speed could be calculated with the known data collection speed of the system and the distance that is defined by the sensitivity matrix. The result will be shown and analyzed in the next section.

## 4. Discussion

In this section, all of the results of both 2D and 3D experimental tests, including the reconstructed images and results of velocity are summarized. The quality of the images are discussed and the cross-correlated results using the proposed spatiotemporal TV algorithms are presented, where the relative errors of the speed are calculated, and further analysis are carried out.

Table 2 shows the speed results. The cross correlated average angular speed is given by the expression 360°/(Transit time × System speed). The speed in this section is the angular speed due to the circular movement of the plastic bar, while the measured time of the whole process, which is 10.8 s and stands for the length of time that the inclusion moves through the whole circle (360°) was determined by a timer, and then the real average angular speed could then be calculated with the formula 360°/(Measured time × System speed). 

In the 3D experiment, as shown in Table 3, the distance used for calculations was the distance between the top voxel layer and the bottom voxel layer, which is defined as 13 cm. It was calculated that the transit time was 17.85 s from the number of frames given by the peak of the cross-correlation plot. The cross-correlated speed and real speed are given by 0.76 and 0.73, respectively, which gives a 4% relative error. 

The 2D and 3D images have been shown in the previous section. It worth noticing that the reconstruction results from simulation and experiment might be different. It needs to be pointed out that the dynamic model is more complicated than a static one as the sample is actually kept moving at any moment, which would affect the data measurement. In the simulation part of both, it was assumed that the boundary data corresponds to a specific location of the sample although what has been simulated is a dynamic model. However, for a real dynamic situation, the location of the sample keeps changing while that frame of data was being measured, which leads to the circumstance that the data are overlapping.

In terms of the image reconstruction quality, the proposed algorithm has a good performance in the 2D test (shown in Figure 2 and Figure 5). By contrast, the object of the experimental images got slightly overlapped compared to the simulation ones, which could also be for the reason stated above. In the 3D simulation and experiment test, the sample movement could be monitored properly although the ones in the middle area are seems relatively weaker than the ones close to rings. Images produced in experiment are still acceptable despite the inter-frame data generated.

In conclusion, the method of flow measurement using ERT with the proposed algorithm is validated by 2D simulations and experimental tests. For the case using 3D ERT, even if the reconstructed images are not as good as in the 2D case and suffer from the problem of inter-frame data, however, a low speed data collection ERT system were used and the simulation study result still shows good performance. The speed measurement result of experimental test is controlled below 4%, which is still acceptable and proved that the speed measurement using the technique of 2D and 3D ERT with spatiotemporal TV algorithm combined and cross correlation is achievable, and the result could potentially be upgraded with a faster ERT data collection system. The cross-correlated result accuracy is actually determined by the images. The quality of a reconstructed image would be quite important and must be upgraded, which is always a challenging topic to study. In further work, in terms of the proposed algorithm, a more effective parameter selection method could be further studied in order to reconstruct images in a more time-efficient way. To eliminate the negative effect of inter-frame data, the time interval of boundary data collection should be shortened, in this case, the data collection system would be required to be fast enough. 

## 5. Conclusions

Velocity profile measurement using a high speed two-channel 2D ERT and cross-correlation between two separate rings has been investigated for a few decades due to its fast implementation in the industrial field. Since flow is a three-dimensional concept, using 2D ERT to obtain consecutive cross-sectional slices may ignore some occurrences in the flow pipe. In this case, it is desirable to combine the measurement method with a 3D reconstruction technique, so a single multi-plane ERT can provide 3D flow velocity information. With a series of phantom experiments, the measurement method based on 2D ERT was tested and validated, and the speed measurement method based on 3D ERT using a spatiotemporal TV algorithm combined with a voxel-voxel cross-correlation method is proposed. The reconstructed images showed the feasibility of the speed measurement of a dynamic sample in 2D and 3D. The process reconstruction of 2D shows great performance with the proposed algorithm. A slightly degraded result in 3D experiments was shown, and the reasons, as stated in the Discussion section, could be due to both insufficient time resolution and the inter-frame data generation. Noise produced in the experiments can also be an influencing factor to degrade the image quality. In this paper, all the experiments were conducted by using an experimental tank with a manually moved inclusion instead of real two-phase flow, so the test is done in a semi-dynamic setting, making it much more robust compared to fully static tests and making the presented results a very useful method for flow measurement and interior flow system monitoring.

## Figures and Tables

**Table 1:** The illustration of current injection pattern and voltage measurement pattern: 16 times of excitations (EX1 to EX16) for 16 pairs of electrode combinations ((1, 2), (2, 3)…… (16, 1)) of the neighboring method are applied. In the table, the current injection pattern marked with **I**, and voltage pattern marked with ‘-’ (× means useless data, that will be removed afterwards).

	EX1	EX2	EX3	EX4	EX5	EX6	EX7	EX8	EX9	EX10	EX11	EX12	EX13	EX14	EX15	EX16
(1, 2)	I	×	-	-	-	-	-	-	-	-	-	-	-	-	-	×
(2, 3)	×	I	×	-	-	-	-	-	-	-	-	-	-	-	-	-
(3, 4)	-	×	I	×	-	-	-	-	-	-	-	-	-	-	-	-
(4, 5)	-	-	×	I	×	-	-	-	-	-	-	-	-	-	-	-
(5, 6)	-	-	-	×	I	×	-	-	-	-	-	-	-	-	-	-
(6, 7)	-	-	-	-	×	I	×	-	-	-	-	-	-	-	-	-
(7, 8)	-	-	-	-	-	×	I	×	-	-	-	-	-	-	-	-
(8, 9)	-	-	-	-	-	-	×	I	×	-	-	-	-	-	-	-
(9, 10)	-	-	-	-	-	-	-	×	I	×	-	-	-	-	-	-
(10, 11)	-	-	-	-	-	-	-	-	×	I	×	-	-	-	-	-
(11, 12)	-	-	-	-	-	-	-	-	-	×	I	×	-	-	-	-
(12, 13)	-	-	-	-	-	-	-	-	-	-	×	I	×	-	-	-
(13, 14)	-	-	-	-	-	-	-	-	-	-	-	×	I	×	-	-
(14, 15)	-	-	-	-	-	-	-	-	-	-	-	-	×	I	×	-
(15, 16)	-	-	-	-	-	-	-	-	-	-	-	-	-	×	I	×
(16, 1)	×	-	-	-	-	-	-	-	-	-	-	-	-	-	×	I

**Table 2:** Calculated results of the 2D experiment.

Measured Time (s)	System Speed (s/frame)	Transit Time from Cross Correlation (s)	Angle (°)	Measured Speed (°/s)	Cross Correlated Speed (°/s)	**Relative Error (%)**
10.80	0.21	10.71	360	33.33	34.29	0.8%

**Table 3:** Calculated results of the 3D experiment.

Measured Time (s)	System Speed (s/frame)	Transit Time from Cross Correlation (s)	Distance (cm)	Measured Speed (cm/s)	Cross Correlated Speed (cm/s)	Relative Error (%)
33.07	0.21	17.85	13.00	0.76	0.73	4%
